# Comparison of semiautomated tangential VMAT with 3DCRT for breast or chest wall and regional nodes

**DOI:** 10.1002/acm2.12442

**Published:** 2018-08-19

**Authors:** Mikel Byrne, Ben Archibald‐Heeren, Yunfei Hu, Andrew Fong, Leena Chong, Amy Teh

**Affiliations:** ^1^ Radiation Oncology Centres Sydney Adventist Hospital Wahroonga NSW Australia; ^2^ Centre of Medical Radiation Physics University of Wollongong Wollongong NSW Australia; ^3^ Radiation Oncology Centres Gosford NSW Australia; ^4^ Sydney Adventist Hospital Clinical School Sydney Medical School University of Sydney Sydney NSW Australia

**Keywords:** breast, nodes, optimization, tangential, VMAT

## Abstract

Radiotherapy to the breast after surgery sometimes requires adjoining nodes to be included in the treatment volume. In these cases, the traditional approach has been a complex 3‐Dimensional Conformal Radiotherapy (3DCRT) beam arrangement which can result in significant dose heterogeneity at the beam junctions. A Volumetric Modulated Arc Therapy (VMAT) beam arrangement has previously been proposed for breast cases, where the chest wall/breast is treated with a limited angle (partial arc) tangential VMAT technique (Virén et al. [2015] *Radiat Oncol*. **10**:79). In our study, this approach is extended to breast and chest wall cases with adjoining nodes by adding a separate conventional VMAT arc field specifically limited to the superior nodes. This VMAT method was implemented using a semiautomated approach on 27 patients, and the resultant plan compared to a monoisocentric 3DCRT plan. Plan statistics, Dose‐Volume Histogram (DVH) analysis and Radiation Oncologist (RO) preference were assessed. When compared to the 3DCRT technique, the VMAT planning method was found to result in better target volume coverage, high doses to organs at risk (OAR) were reduced but greater OAR volumes received low doses. Having said that, the volume receiving low doses with this tangential VMAT technique was less than that of other VMAT planning methods described in the literature, and the integral dose was less than the 3DCRT method. The VMAT technique also resulted in more robust junction doses that the 3DCRT method. RO review found that the VMAT technique was preferred in 81% of cases. Specifically, the VMAT plans were preferred in all categories of patients except left chest wall cases where the intermammary nodes were also treated. The VMAT technique described here is a useful addition to the treatment options available for breast/chest wall and nodal patients.

## 1 INTRODUCTION

Radiotherapy to the breast or chest wall is commonly given as an adjuvant treatment after breast cancer surgery. In some cases where nodal spread is suspected or confirmed, the supraclavicular (SC), axillary (AX), and/or the internal mammary (IM) nodes are also treated. In these cases, the traditional standard treatment has been the combination of opposed tangential 3‐Dimensional Conformal Radiotherapy (3DCRT) beams, sometimes with additional subfields at the same gantry angles, junctioned with anterior‐posterior 3DCRT beams to the AX and SC nodes, and in some cases electrons to the IM nodes. Variations on this technique exist, but all 3DCRT techniques require forward planned field junctions in the regions between the breast/chest wall PTV and the nodal PTVs. Numerous other methods exist to treat these cases including electronic compensation,^1^, ^2^ where the MLCs are moved dynamically according to the tissue separation at each location. Some research^3^, ^4^, ^5^ has suggested that inversely optimized techniques, such as Volumetric Modulated Arc Therapy (VMAT), may improve coverage while reducing organ at risk (OAR) doses in this scenario.

However, the research on VMAT breast planning thus far has found that it results in a larger area of low dose in the patient^3^, ^5^, ^6^, ^7^, ^8^, ^9^, ^10^; specifically contralateral breast, contralateral lung and heart receive low doses to a volume far greater than would be delivered from traditional 3DCRT breast treatments. This low‐dose bath to healthy organs has been associated with increased risk of secondary malignancies^11^, ^12^ and adverse cardiac events.^13^ A VMAT technique has been proposed that reduces this issue by using tangential VMAT partial arcs for treatment of the breast only.^4^, ^15^ This has been shown to reduce the volume of the patient receiving a low dose while maintaining good target coverage. However, the method as described does not allow for treatments to adjacent regional nodes, which are likely to benefit most from the use of VMAT, due to their complex shapes and proximity to OARs. The authors hereby propose a VMAT technique for complex breast cases that require regional nodal irradiation that combines tangential VMAT arcs for treatment of the breast/chest wall, with a separate conventional VMAT arc that is specifically limited to the AX and SC nodes and any junction region. This method has the potential to get the greatest benefits from VMAT by; (1) utilizing tangential arcs for the breast/chest wall PTV thus reducing the low dose region when irradiating the chest, (2) using full arc VMAT to maximize coverage and OAR avoidance when irradiating the AX and SC, and (3) improving dose homogeneity across the field junction. If this method achieves comparable low‐dose spill to 3DCRT methods, this would diminish concerns about the significance of low‐dose spill from VMAT relative to 3DCRT in breast cases.^9^, ^16^ This technique also has the potential benefit of being quicker to plan compared to the 3DCRT technique.

In the RayStation v5.0 treatment planning system (RaySearch Laboratories, Stockholm, Sweden), this tangential VMAT planning method can be partially automated through the use of relatively simple scripting. The automation allows the creation of plans, placement of beams, selection of optimization settings, and optimization. This allows for faster and more consistent treatment planning.^17^ This scripted method can also be combined with manual optimization, tailored to the patient's specific anatomy to finish the plan, described here as a semiautomated planning approach.

Respiratory motion presents two potential challenges for inverse planned dynamic fields used in breast radiotherapy^18^; ensuring coverage on the patient external edge with motion, and the minimization of interplay effects. Coverage on the patient external was ensured in this case using the RayStation robust optimization feature,^19^ but can equally well be dealt with using virtual bolus.^20^ Interplay is generally not considered an issue if the motion is less than 1 cm^21^ and many fractions are used.^22^ Both of these conditions are met in conventionally fractioned breast treatments.^18^, ^23^, ^24^

This study presents a plan comparison of the semiautomated combined VMAT technique versus the conventional 3DCRT technique on breast and chest wall cases with adjacent nodal volumes. Plan statistics and Radiation Oncologist (RO) evaluation of the plans were used to determine the preferred plan in each case.

## 2 METHOD

The 30 most recently treated breast or chest wall radiotherapy cases that also required regional nodal irradiation from the authors institution were selected. Each case was checked to ensure that each treated region had a clearly labeled and unique CTV and corresponding PTV. After eliminating unsuitable cases, 27 cases remained representing a variety of breast/chest wall and nodal regions being irradiated. All cases selected had at least one nodal region irradiated, and in most cases numerous nodal regions irradiated. Eight of the patients were left‐sided, while 19 were right‐sided. The cohort included both patients that had received breast conserving surgery (12) as well as mastectomy (15). None of the patients utilized the deep inspiration breath hold (DIBH) technique during scan or treatment. These cases had previously been treated using a 3DCRT conventional planning technique and were replanned using the tangential VMAT technique in the RayStation v5.0 planning system. The prescription used in this study was 50 Gy in 25 fractions for all patients. The linear accelerator used for all plans was a Varian 21iX with millennium MLC.

### 2.A Planning

#### 2.A.1 3DCRT Planning

The 3DCRT plans were manually created and planned. Within this cohort, the gantry and collimator angles were not strictly limited, but rather chosen to maximize plan quality for the specific anatomy of the patient. However, they followed a basic formula; the plans were monoisocentric with open parallel opposed tangential beams for the breast/chest wall, in combination with forward planned segments where required at the same gantry angles designed to maximize coverage and minimize any hotspots within the breast/chest wall PTV. These were then junctioned with parallel opposed fields for the SC and AX region. Each of the beams was shaped with the MLC. The plans included both 6 and 18 MV beams, as selected by the treatment planner, although in most cases (25/27) both 6 and 18 MV was used. In a number of the plans (5), electrons were junctioned with the photon fields, usually to cover IM nodes. A number of the plans also used physical bolus. In cases where bolus was used, the corresponding VMAT plan also used the same bolus. Note that all the 3DCRT plans were previously approved and clinically treated plans.

#### 2.A.2 VMAT planning

The VMAT planning method utilized the tangential VMAT method described by Virén et al.^1^ This beam arrangement was combined with an approximately 240° arc used to treat the AX and SC PTVs. A single isocenter was used for all arcs which was positioned in the geometric center of all the PTVs combined, usually falling close to the chest wall toward the superior edge of the breast or chest wall PTV. The tangential arcs were set to the breast/chest wall PTV using the jaw limits in the “beam optimization settings” in RayStation. Similarly, the AX and SC arc was set to the AX and SC PTVs using the same settings. To allow for more efficient gantry travel during delivery, the AX/SC arc was split in two at the location of the lateral breast/chest wall arcs. Where the breast/chest wall PTVs were contiguous with the AX/SC PTVs, an overlapping junction region of 2–3 cm was allowed, where both sets of arcs were able to treat the PTV in this region. This allowed for the junction to be handled by the optimizer in a gradual way such that the plan was robust to small differential positioning offsets.

The planning and plan evaluation was performed using a semiautomated workflow. The semiautomated planning workflow added planning volumes required automatically, then created the plan, added the isocenter, selected beam and collimator angles, added objectives and clinical goals. The energy used in each VMAT plan was 6 MV. The collimator angles used were designed to minimize MLC travel, and were consistent for each laterality. The tangential VMAT beam angles were found by determining the location of both the medial and posterior extent of the PTV, then finding the angle between these points to give the nominal tangent angle. The tangential VMAT gantry range was then set;

- For the medial arcs as a 40° range starting 30° inside the nominal tangent angle and continuing 10° outside. This offset range was chosen to minimize the travel of the arc across the patient midline.
- For the lateral arcs as a 50° range starting 20° inside the nominal tangent angle and continuing 30° outside.

**Figure 1:**
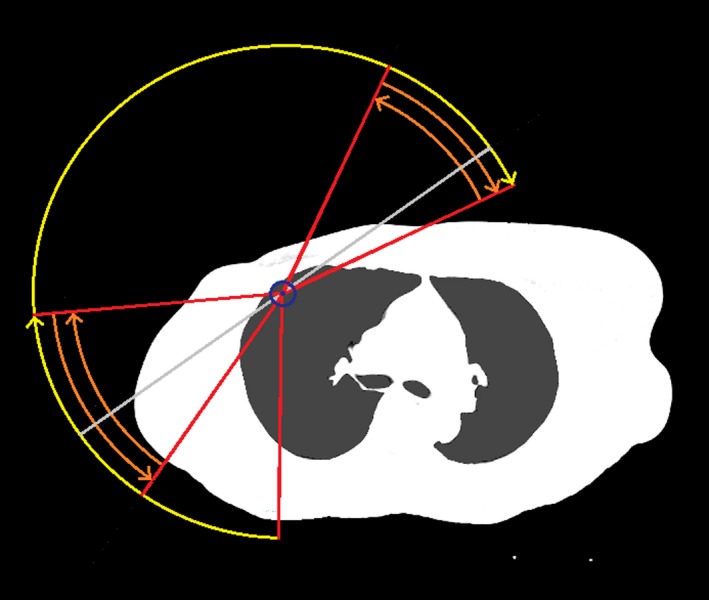
Schematic illustration of an example of the VMAT arc gantry ranges used for a right breast case. The arcs are separated longitudinally with the four short arcs (orange) only used to treat the breast/chest wall and IM PTVs and junction region, while the longer arcs (yellow) are used for the AX and SC PTVs and junction region. The nominal tangent angle determined by the script is shown as a light gray line.

These arc ranges were chosen based on the recommendations of Virén et al.,^1^ and preliminary testing within the RayStation system. The RayStation dual arcs function was used to allow the gantry to pass each location twice in the breast/chest wall region and provide better coverage. See Fig. 1 for an example of the arc ranges.

At this point, the planner manually adjusted the maximum jaw positions allowed, so as to cover the correct volumes, as this cannot be scripted in RayStation v5.0. A second script was then run that linked each clinical goal with an optimization objective. The script then optimized the plan for 50 iterations, checked the clinical goals, for any goal that was not met, increased the weighting of the optimization objective linked to the failed goal, then continued the optimization. This optimization method was described by Archibald‐Heeren et al.^17^ This was continued for a total of 300 iterations, after which the plans were manually reviewed and further optimized if thought necessary.

### 2.B Plan evaluation

The dose‐volume histogram (DVH) parameters evaluated (Table 1) were loosely based on the RTOG 1304 clinical trial^25^ and used a prescription of 50 Gy in 25 fractions to the breast/chest wall and nodal regions.

**Table 1:** The DVH parameters used in the VMAT planning based on RTOG 1304 clinical trial for different regions of interest (ROI).^25^

ROI	Plan metric	Acceptable value	Ideal value
Target volume coverage and dose homogeneity			
PTV Breast/Chest wall	V90 (V45 Gy)	>90%	>99%
PTV Breast/Chest wall	D95	>90%	>95%
PTV Breast/Chest wall	D0.3 cc	<120%	<115%
PTV IM	V90 (V45 Gy)	>90%	>99%
PTV IM	D95	>90%	>95%
PTV IM	D0.03 cc	<115%	<110%
PTV AX	V90 (V45 Gy)	>90%	>99%
PTV AX	D95	>90%	>95%
PTV AX	D0.03 cc	<115%	<110%
PTV SC	V90 (V45 Gy)	>90%	>99%
PTV SC	D95	>90%	>95%
PTV SC	D0.03 cc	<115%	<110%
External	D0.03 cc	<120%	<110%
Organs at risk dose constraints			
Lung (Ipsilateral)	V20 Gy	<35%	<15%
Lung (Ipsilateral)	V10 Gy	<60%	<50%
Lung (Ipsilateral)	V5 Gy	<70%	<65%
Lung (Contralateral)	V5 Gy	<15%	<10%
Heart	Mean dose	<500 cGy	<400 cGy
Heart	V25 Gy	<10%	<5%
Heart	V15 Gy	<15%	<10%
Heart	D0.03 cc	<3000 cGy	<2500 cGy
Contralateral breast	D0.03 cc	<1000 cGy	<300 cGy
Contralateral breast	D5	<410 cGy	<300 cGy
Spinal cord	D0.03 cc	<4500 cGy	<4000 cGy

The plans were blinded and separately reviewed by three ROs. Each RO selected the plan that they thought was clinically superior. During this process, the ROs were told not to assess the accuracy of contouring, to remove any contouring differences from the plan analysis as much as possible. To simplify the process, ROs were not given access to the patient history, demographics or notes, and therefore were forced to make some assumptions about the priority of treating the nodes relative to sparing healthy tissue. To blind the RO to the planning technique, the beam displays were disabled, so that all that was visible to the RO during plan review was the patient CT and the two plans’ dose distributions.

## 3 RESULTS

The plan analysis and comparison based on dose‐volume factors are summarized in Table 2. It was found that a number of the plan parameters were not normally distributed and therefore nonparametric statistics were used for analysis. The location parameter used is the Hodges‐Lehmann estimator, referred to as the pseudo‐median, with 95% confidence intervals calculated using Walsh averages. The Wilcoxon signed rank test was used to determine statistical significance, with the null hypothesis (H_0_) that there is no difference between the planning techniques, and statistical significance set at *α* = 0.05.

**Table 2:** Target volume coverage and OAR dose metrics achieved for 3DCRT and VMAT planning methods

ROI	Plan metric	3DCRT			VMAT			Superior technique	Accept or reject H_0_
		Pseudo‐median (Hodges‐Lehmann)	95% confidence interval	Clinical goal	Pseudo‐median (Hodges‐Lehmann)	95% confidence interval	Clinical goal		
Target volume coverage and dose homogeneity									
PTV breast/chest wall	V90 (V45 Gy)	98.1%	(87.5%–99.8%)	Acceptable	99.3%	(97.2%–99.9%)	Ideal	VMAT	Reject
PTV breast/chest wall	D95	93.6%	(77.5%–96.9%)	Acceptable	96.5%	(93.1%–98.6%)	Ideal	VMAT	Reject
PTV breast/chest wall	D0.3 cc	109.9%	(106.3%–122.9%)	Ideal	108.4%	(107.3%–111.1%)	Ideal	VMAT	Reject
PTV breast/chest wall	V105 (V52.5 Gy)	11.0%	(1.1%–25.8%)	NA	12.5%	(7.3%–22%)	NA	3DCRT	Accept
PTV IM	V90 (V45 Gy)	92.3%	(51.5%–99.2%)	Acceptable	96.8%	(89.2%–98.3%)	Acceptable	VMAT	Accept
PTV IM	D95	89.3%	(36.3%–93.7%)	Fail	92.8%	(84.1%–94.5%)	Acceptable	VMAT	Accept
PTV IM	D0.03 cc	106.6%	(101.1%–111%)	Ideal	108.3%	(107.6%–109.7%)	Ideal	3DCRT	Accept
PTV AX	V90 (V45 Gy)	98.0%	(94.8%–100%)	Acceptable	99.7%	(99.1%–100%)	Ideal	VMAT	Accept
PTV AX	D95	94.0%	(89.8%–98.7%)	Acceptable	97.5%	(95.8%–99.4%)	Ideal	VMAT	Reject
PTV AX	D0.03 cc	107.1%	(104.8%–110.7%)	Ideal	108.0%	(107.1%–109.1%)	Ideal	3DCRT	Accept
PTV SC	V90 (V45 Gy)	98.3%	(78.4%–100%)	Acceptable	99.9%	(98.9%–100%)	Ideal	VMAT	Reject
PTV SC	D95	93.4%	(55.2%–97.8%)	Acceptable	98.0%	(95.2%–99.3%)	Ideal	VMAT	Reject
PTV SC	D0.03 cc	104.8%	(102.2%–109.8%)	Ideal	107.6%	(106.6%–109.1%)	Ideal	3DCRT	Reject
External	D0.03 cc	110.3%	(106.6%–122.9%)	Acceptable	108.6%	(107.4%–111.9%)	Ideal	VMAT	Reject
Organs at risk dose constraints									
External	V5 Gy (cc)	4509.3	(2691.8–6596.9)	NA	5444.7	(3432.8–7483.6)	NA	3DCRT	Reject
External	D_integral_ (Gy x L)	176.1	(107.7–259.1)	NA	165.8	(97.6–232.4)	NA	VMAT	Reject
Lung (Ipsilateral)	V20 Gy	27.2%	(15.7%–42%)	Acceptable	22.3%	(15%–33.9%)	Acceptable	VMAT	Reject
Lung (Ipsilateral)	V10 Gy	35.2%	(20.5%–48.8%)	Ideal	37.4%	(24%–50.2%)	Ideal	3DCRT	Accept
Lung (Ipsilateral)	V5 Gy	46.0%	(28.9%–59.9%)	Ideal	55.5%	(38.9%–67%)	Ideal	3DCRT	Reject
Lung (Contralateral)	V5 Gy	0.0%	(0%–0.6%)	Ideal	2.0%	(0%–8.1%)	Ideal	3DCRT	Reject
Heart	Mean dose (cGy)	100.9	(42.8–401.7)	Ideal	192.2	(90.5–592.0)	Ideal	3DCRT	Reject
Heart	V25 Gy	0.1%	(0%–4.8%)	Ideal	0.0%	(0%–4.4%)	Ideal	VMAT	Accept
Heart	V15 Gy	0.3%	(0%–7.2%)	Ideal	0.2%	(0%–10.1%)	Ideal	VMAT	Accept
Heart	D0.03 cc (cGy)	1176.4	(375.3–3206.9)	Ideal	1255.4	(518.1–2408.2)	Ideal	3DCRT	Accept
Contralateral breast	D0.03 cc (cGy)	872.8	(204.6–4719.6)	Acceptable	848.1	(379.8–1756.7)	Acceptable	VMAT	Accept
Contralateral breast	D5	130.4	(62.2–334.5)	Ideal	309.0	(163.7–515.0)	Acceptable	3DCRT	Reject
Spinal cord	D0.03 cc (cGy)	1339.4	(147.4–3872.1)	Ideal	1456.0	(467.6–2570.4)	Ideal	3DCRT	Accept

These results show that the VMAT technique had statistically superior target coverage for the breast/chest wall, AX, and SC PTVs. The results for the IM PTV were not statistically significant, due to the relatively small patient numbers (11), and large variation in coverage in the cohort. The OAR results were more mixed, generally with VMAT superior at the higher dose metrics and 3DCRT superior at the lower dose metrics.

**Figure 2:**
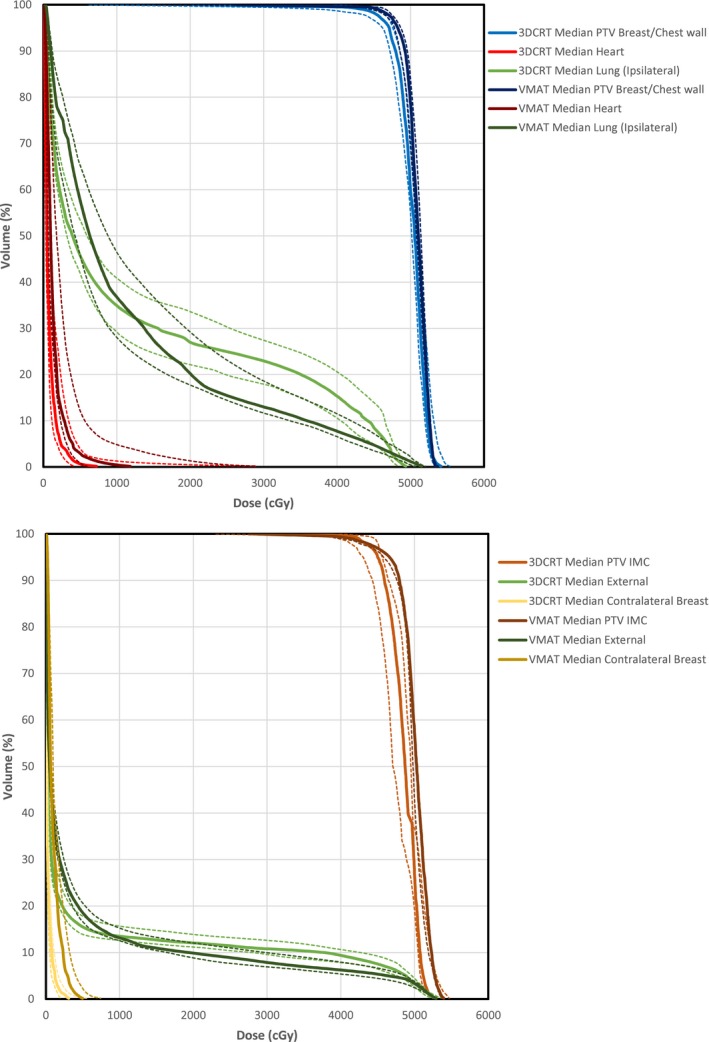
The median DVH for both 3DCRT and VMAT planning methods shown for selected regions of interests with interquartile ranges as dashed lines.

The median DVH for selected PTVs and OARs is shown in Fig. 2. Again, the characteristic differences in the DVH with the planning methods can be seen; VMAT has improved PTV coverage, lower volumes of OARs receiving high doses, and greater volumes of OARs receiving low doses.

The results of the RO plan reviews were; in 10 cases (37%) the VMAT plan was preferred by all three ROs, in 12 cases (44%) the VMAT plan was preferred by two of three ROs, in five cases (19%) the 3DCRT plan was preferred by two of three ROs, and in 0 cases the 3DCRT plan was preferred by all three ROs. It can be seen that the VMAT plans were preferred over the 3DCRT plans in 81% of cases.

The average MU for the 3DCRT and VMAT planning methods were 487 and 728 MU, respectively. Assuming the 3DCRT plan was delivered at the maximum dose rate of 600 MU/min, this leads to an estimated beam‐on time for the 3DCRT plan of 49 s. Using the built‐in RayStation arc duration estimation, the average beam‐on time for the VMAT plan was 112 s. With careful ordering of the VMAT arcs, it is possible to minimize the gantry travel between arcs to equivalent or less than the 3DCRT plan. This leads to a total time from first beam‐on to last beam‐off of roughly 1 min longer for the VMAT plan as compared to the 3DCRT plan (excluding the 3DCRT plans requiring electrons, which are expected to have much longer treatment times due to the electron setup time).

## 4 DISCUSSION

**Figure 3:**
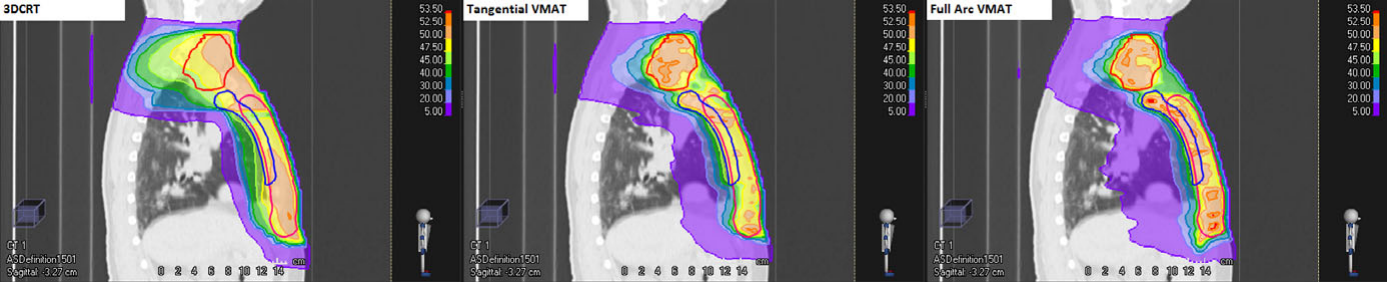
Comparison of typical dose distributions seen from multiple planning methods for a chest wall case with IM, SC and AX nodes. On the left is the clinical 3DCRT plan, in the middle is the tangential VMAT plan, and on the right is a full arc VMAT plan. The lowest isodose line displayed (purple) is 5 Gy to highlight the differences in the volume covered by this isodose.

From Table 2, it can be seen that VMAT was generally better for target coverage, however was marginally worse for a number of OAR metrics. Specifically, VMAT tended to be better at higher OAR dose metrics, but worse for the low‐dose OAR metrics. A range of metrics are used to quantify low‐dose volumes in the literature; in this case, the total volume receiving 5 Gy (V5 Gy) and integral dose (including PTVs) were compared. It can be seen that indeed the volume receiving 5 Gy was significantly higher with the VMAT method as compared to 3DCRT; however, the integral dose using VMAT was significantly less than 3DCRT. The average reduction in integral dose seen with VMAT was 10.8 Gy × L. Additional VMAT imaging requirements are not accounted for in this calculation; however, this differential would more than compensate for even the worst case imaging dose. The use of the tangential VMAT method for the breast/chest wall region has substantially reduced the low‐dose volume when compared to other VMAT techniques in use.^3^, ^5^, ^6^, ^7^, ^10^ A comparison of various low‐dose metrics for different VMAT planning methods is shown in Table 3. To allow comparison with the patient cohorts used in the different studies, data from the present study are shown for all cases and left cases only. The metrics displayed in this table were normally distributed and therefore the average value is reported to better allow comparison with other studies. A single sample two‐tailed t test was performed between the data from this study and the best case planning parameter from any one of the other studies with comparable cohort listed in the table, to determine whether the reduction in low‐dose metrics was significant. In each case *P* < 0.001, indicating there was a significant reduction in the low‐dose metrics. This significant reduction in low‐dose spill makes the tangential VMAT technique more comparable to 3DCRT in terms of low‐dose metrics. The typical features of the 3DCRT and tangential VMAT dose distributions are shown in the sagittal view in Fig. 3. A full arc VMAT plan with optimization parameters identical to the tangential VMAT plan, and beam arrangement according to the method described by Tyran et al.^3^ is also shown in Fig. 3 for illustrative purposes. Differences in the penetration of the 5 Gy isodose line into the ipsilateral lung can be seen between the planning methods.

**Table 3:** Comparison of common low‐dose metrics for a range of VMAT studies. Laterality indicated in brackets refers to the treatment plan. ^1^Bowan et al. report on several different arc ranges but metrics for the 240° dual arc without arc splitting (NS240) are reported, as this arrangement was generally the best for contralateral lung and contralateral breast

Study	Patient cohort	Plan method	Number of patients	Prescription (Gy)	Low‐dose metric				
					External V5 Gy (cc)	Lung (Contralateral) mean dose (Gy)	Lung (Contralateral) V5 Gy (%)	Heart mean dose (Gy)	Contralateral breast mean dose (Gy)
Present study	Lt and Rt + various nodes	As described	27	50	5444	0.9	2.4	2.4	1.1
Present study (only Lt patients)	Lt + various nodes	As described	8	50	6057	1.0	3.7	5.3	1.5
Tyran et al.^2^	Lt + all nodes	240° (dual)	10	50		4.0	15.0^a^	8.6	3.2
Boman et al.^4^	Lt and Rt + various nodes	240° (dual)¹	19	50	9534^a^	3.9 (Lt), 2.6 (Rt)	24.1 (Lt), 5.4 (Rt)	7.7 (Lt), 4.6 (Rt)	4.1
Badakhshi et al.^5^	Lt and Rt + SC nodes in 4 cases	360°	12	50		5.6 (Lt)	19.5	12.4 (Lt)	
Johansen et al.^6^	Lt and Rt + all nodes	361°	8	50		2.9^a^		4.6^a^	2.0^a^
Pasler et al.^21^	Lt breast + SC nodes	Approx. 250° (single)	10	50		3.4	23.0	8.9	2.8

a Indicates that these data were used in single sample t test comparison to the present study.

In 22/27 cases, the VMAT plan was preferred by the majority of ROs, and in 5/27 the 3DCRT plan was preferred. Of the five patients where the 3DCRT plan was preferred to the VMAT plan by the majority of ROs, three had very similar anatomy and plan characteristics not shared with the rest of the cohort. The specific characteristics shared by these plans were that the left chest wall and IM nodes were among the volumes being treated, and the heart was adjacent to the treatment area. In each of these cases, the 3DCRT plan had an electron field that covered the medial chest wall PTV and IM nodes and had been junctioned with the tangential photon field, giving good sparing of the heart. For these specific patients, the VMAT technique could not match the sparing of OARs from the traditional 3DCRT‐based technique, although it generally did give better coverage. These were the only plans included in the study that had the combination of left chest wall, IM nodes and a clinical plan that utilized electrons. Note that during the plan review, the ROs were told to assume that the dose distribution would be delivered to the patient as seen on screen, however for cases with a photon‐electron junction like these, there is likely greater setup uncertainty in the field junction region than for the other techniques utilized. Of the remaining two patients where the 3DCRT plan was preferred by the majority, discussions with the ROs suggested that in these cases both plans were considered almost equivalent.

In the 10 plans where VMAT was unanimously preferred, VMAT both increased coverage and reduced OAR doses and the RO choice was easy. Within the remaining group of plans, more trade‐offs were seen, where the VMAT plan was not better in all areas. It was noted that one RO tended to prioritize improved coverage and reduced maximum dose, while the other two prioritized improved OAR sparing in these trade‐off areas. This is probably due to the somewhat vague instructions given to the ROs about the assumptions to make about the patients. An assumption of confirmed positive nodes would change the priority of the coverage of the nodes relative to the OARs. In no cases was the 3DCRT plan unanimously the preferred option.

One of the limitations of the semiautomated method used to create the VMAT plans is that they were planned very much according to the clinical goals. In cases where the clinical goal was easily met, the optimizer did not reduce the goal further despite in some cases further improvement being possible. This difference can particularly be seen in how many of the clinical goals were passed at the ideal level by the pseudo‐median VMAT plan (19/24) as compared to the pseudo‐median 3DCRT plan (13/24). Of the five plan metrics where there was a statistically significant difference between the plans and the 3DCRT plan was better, in four of them the VMAT plan had already met the ideal goal and therefore was not working to improve the plan in this area.

One of the advantages of using 40°–50° arcs for the breast/chest wall is that the precise selection of gantry and collimator angles is far less critical. The method of selecting nominal tangential gantry angles did not always closely agree with the choice of tangential gantry angles from the manual 3DCRT plan; however due to the use of short arcs, these differences were partially compensated for in the optimization process. It is noted that if the planning system offered the option of directionally blocking parts of an arc to parts of the PTV, it potentially would be possible to deliver the entire VMAT beam arrangement described here in a single arc rotation.

**Figure 4:**
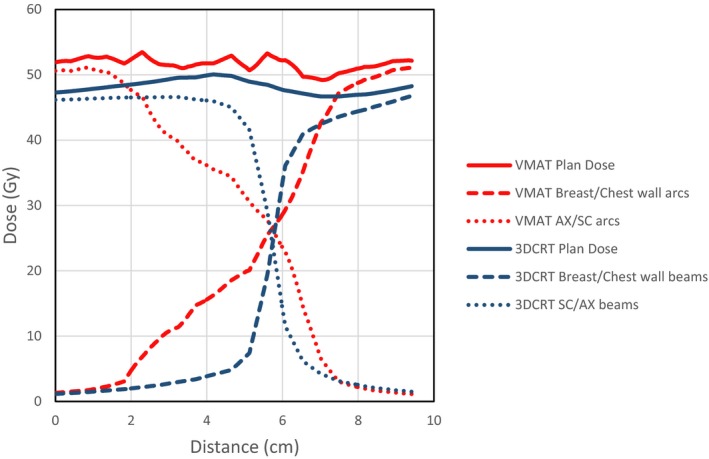
Dose contribution across junction region between breast/chest wall and AX/SC nodes from respective beams/arcs for 3DCRT and VMAT plans for an example case.

**Figure 5:**
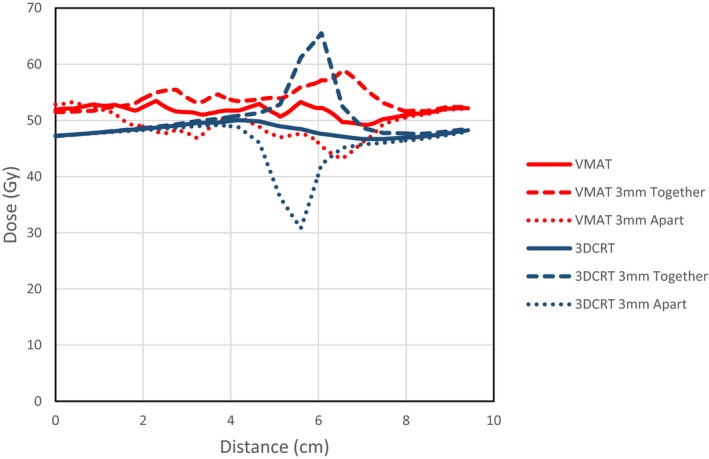
Dose change across junction region with 3 mm longitudinal shift of SC/AX beams relative to the breast/chest wall beams for 3DCRT and VMAT plans for an example case.

The planning technique covers the junction region with both the AX/SC arcs and the breast/chest wall tangential arcs. With this beam arrangement, the optimizer automatically ensures that the dose is feathered across this region, and that the plan is robust to small interfraction movements. This occurs because the optimization algorithm starts the optimization with the fluence set to the projection of the target at each gantry angle, thus splitting fluence between the beams that cover the junction region.^26^ The fluence is then modified from this initial configuration during the optimization process, but always maintains some fluence through each of the beams in this region, thus making the junction dose more gradual than the hard junction seen in 3DCRT plans. This overlap region was a minimum of 2 cm in the superior‐inferior direction, but due to collimator angles could be larger in some areas. A representative example of the overlap region for one of the VMAT plans is shown in Fig. 4, displaying the gradual dose gradients. This line dose was taken at an oblique angle along the chest wall, hence the larger junction region seen. This example was recalculated with the AX/SC beams and arcs offset by 3 mm longitudinally, to simulate differential interfraction motion. The expected dose variations in this scenario can be seen in Fig. 5. While the VMAT has a larger region with dose variation, the magnitude of the dose variation is significantly reduced. Note that Fig. 5 shows the dose distribution resulting if the whole treatment course was delivered with this differential motion, in practice these differences average over numerous fractions.

Although not explicitly tested in this study, the time taken to plan these patients using the semiautomated VMAT method is expected to be substantially quicker than the 3DCRT planning method. The automated plan creation component took approximately 30 min to complete, which then required approximately 1 hr of further optimization to result in a finished plan. This is longer than would normally be expected for RayStation optimization and is probably due to the use of robust optimization, which can take up to three times longer per iteration.^19^ Note that this plan timing does not include the anatomy contouring step, which was not part of this study.

While all attempts were made to blind the RO from the planning method used during plan evaluation, the dose distributions differ significantly with characteristic shapes, making it possible for the RO to determine the planning method based on the dose distribution alone. It is acknowledged therefore that it is possible that the RO occasionally knew which plan they were selecting as the preferred option.

Another study limitation is that in practice PTV contouring can vary according to the treatment planning method being used. As the patients used in the study were contoured with the RO expecting the treatment plan to be 3DCRT, in areas where coverage would easily be achieved it is possible they implicitly assumed the coverage would be adequate and paid less attention to PTV contouring in these areas. However when these PTVs are used with a different planning method, in this case VMAT, these implicit assumptions about coverage may not hold true and therefore may result in inadequate coverage in some areas. This was noted during plan review by ROs on two occasions, but if anything tended to favor the 3DCRT plan.

The results suggest that the VMAT planning method results in superior plans for the majority of patients. The results also indicate that for a small subset of patients; left chest wall patients where the IM nodes require treatment and heart is close to the chest wall, this planning method gives inferior results when compared to existing planning methods. This planning scenario is particularly complex, and neither plan for these patients was ideal. It is likely that the use of DIBH in this scenario would substantially improve both plans and may lead to more favorable results for the VMAT method, although this may introduce other motion‐related considerations.

While it is often possible to get good dose distributions in the planning system, how these translate into delivered dose distributions can vary, due to inter and intrafraction motion both near the breast/chest wall field junction, and on the patient external edge. The results indicate that the VMAT technique described has the potential to result in improved dose distributions while taking recommended precautions to account for patient movement. The usability results indicate that the VMAT technique may increase the average treatment time by approximately 1 min.

## 5 CONCLUSION

Treatment planning of the breast/chest wall with nodes presents a very complex treatment planning situation. The results of this study indicate that while there are advantages and disadvantages to both 3DCRT and VMAT treatment plans in this scenario, overall the VMAT plans were generally better. The combination of tangential VMAT arcs for the breast/chest wall with a larger arc for the AX and SC nodes as described above maintains many of the advantages of VMAT planning, while minimizing the volume receiving low doses, particularly on the contralateral side of the patient. In particular, this tangential VMAT method resulted in significantly lower integral dose to the patient than the 3DCRT planning method. This planning procedure can be carried out quickly and easily with a semiautomated approach using scripting functions already available in the clinic.

Based on these results, the planning method utilized for any given patient should be decided on a case by case basis, determined by the patients treatment requirements and anatomy. However, the availability of the VMAT technique described here is a useful addition to the treatment options available for this difficult planning situation.

## CONFLICTS OF INTEREST

The authors declare no conflicts of interest.
